# Spinal cord dorsal horn sensory gate in preclinical models of chemotherapy-induced painful neuropathy and contact dermatitis chronic itch becomes less leaky with *Kcc2* gene expression-enhancing treatments

**DOI:** 10.3389/fnmol.2022.911606

**Published:** 2022-11-24

**Authors:** Michele Yeo, Qiaojuan Zhang, LeAnne Ding, Xiangjun Shen, Yong Chen, Wolfgang Liedtke

**Affiliations:** ^1^Departments of Neurosurgery, Duke University Medical Center, Durham, NC, United States; ^2^Departments of Neurology, Duke University Medical Center, Durham, NC, United States

**Keywords:** spinal cord dorsal horn, *Kcc2* = potassium-chloride cotransporter 2, TRPV4, TRPA1, kenpaullone, pain, itch (pruritus)

## Abstract

Low intraneuronal chloride in spinal cord dorsal horn (SCDH) pain relay neurons is of critical relevance for physiological transmission of primary sensory afferents because low intraneuronal chloride dictates GABA-ergic and glycin-ergic neurotransmission to be inhibitory. If neuronal chloride rises to unphysiological levels, the primary sensory gate in the spinal cord dorsal horn becomes corrupted, with resulting behavioral hallmarks of hypersensitivity and allodynia, for example in pathological pain. Low chloride in spinal cord dorsal horn neurons relies on the robust gene expression of *Kcc2* and sustained transporter function of the KCC2 chloride-extruding electroneutral transporter. Based on a recent report where we characterized the GSK3-inhibitory small molecule, kenpaullone, as a *Kcc2* gene expression-enhancer that potently repaired diminished *Kcc2* expression and KCC2 transporter function in SCDH pain relay neurons, we extend our recent findings by reporting (i) effective pain control in a preclinical model of taxol-induced painful peripheral neuropathy that was accomplished by topical application of a TRPV4/TRPA1 dual-inhibitory compound (compound 16-8), and was associated with the repair of diminished *Kcc2* gene expression in the SCDH; and (ii) potent functioning of kenpaullone as an antipruritic in a DNFB contact dermatitis preclinical model. These observations suggest that effective peripheral treatment of chemotherapy-induced painful peripheral neuropathy impacts the pain-transmitting neural circuit in the SCDH in a beneficial manner by enhancing *Kcc2* gene expression, and that chronic pruritus might be relayed in the primary sensory gate of the spinal cord, following similar principles as pathological pain, specifically relating to the critical functioning of *Kcc2* gene expression and the KCC2 transporter function.

## Introduction

Spinal cord dorsal horn (SCDH) neuronal chloride is established as a critical determinant of sensory circuit integrity (Basbaum, 1999; Kahle et al., 2014; Liang et al., 2015; Liedtke, 2022). Low neuronal chloride in the CNS neurons of vertebrates is maintained by the chloride-extruding KCC2 electroneutral chloride transporter (Doyon et al., 2013; Mapplebeck et al., 2019; Yeo and Liedtke, 2020). A key role of *Kcc2* expression and KCC2 function has been demonstrated in animal physiology and preclinical models for pain, since functional KCC2 maintains chloride at a low concentration in CNS neurons. This guarantees the inhibitory function of ionotropic ion channel receptors for GABA and glycine (Chudotvorova et al., 2005; Fiumelli et al., 2005; Agez et al., 2017; Spoljaric et al., 2019). For stable KCC2 expression and function, which maintains steady chloride ion extrusion from neurons, the following features are key: appreciable gene expression of *Kcc2*, proper post-translational modification of the KCC2 protein and its location to the outer plasma membrane of the neuron. *Kcc2* gene expression is fundamental to all functions of the KCC2 protein, and attenuation of *Kcc2* gene expression in pain relay neurons has been demonstrated as causal and key for chronic pain (Doyon et al., 2013; Gagnon et al., 2013; Kahle et al., 2014; Yeo and Liedtke, 2020; Yeo et al., 2021; Liedtke, 2022), with a clean demonstration of causality in several preclinical models of pathologic pain (Coull et al., 2003; Gagnon et al., 2013; Kahle et al., 2014). Suggestive evidence in human systems stems from observations in spinal circuits using organotypic spinal cultures, derived from early post-mortem material (Dedek et al., 2019).

With regards to *Kcc2* gene expression, the DNA regulatory regions of the *Kcc2* gene were found to be critical, namely REST-RE-1, Egr-EGR4, E-box-USF1/2, and most recently Kaiso sites (Yeo et al., 2013b; Yeo et al., 2021; Liedtke, 2022). Kaiso sites depend on DNA methylation to function as transcriptional regulators, indicative of their key relevance in epigenetic gene regulation. Kaiso binding sites can be critically regulated by binding δ-catenin-KAISO complexes (Prokhortchouk et al., 2001; Lopes et al., 2008; Hong et al., 2010; Kaplun et al., 2021).

We recently reported the results of a delimited screening campaign of compounds that suppress the growth of malignantly transformed cells (“cancer drugs”), screened for their ability to upregulate *Kcc2* gene expression because many of these compounds affect epigenetic regulation (Yeo et al., 2021). Of several advantageous compounds that we identified in our screening and subsequent work-up, kenpaullone was selected for in-depth characterization. We demonstrated that the GSK3β-inhibitory effects of kenpaullone were key for its *Kcc2* gene expression-enhancing effects. The mechanism of action in neurons was an enhanced nuclear transfer of non-phosphorylated δ-catenin, reflective of the inhibited kinase activity of GSK3β, which phosphorylates δ-catenin, by kenpaullone. In the nucleus, δ-catenin bound to KAISO transcription factors, and δ-catenin/KAISO bound to Kaiso DNA binding sites. We discovered two previously unrecognized Kaiso sites directly adjacent to the transcriptional start site of *Kcc2* that regulate *Kcc2* transcription. Kenpaullone was safe and effective as an analgesic in mouse preclinical models of nerve injury and cancerous bone pain, with a protracted, yet long-lasting duration of analgesic action. This observation is compatible with an effect on the gene expression of *Kcc2*, not the direct and rapid effect on chloride extrusion by the KCC2 transporter protein, further supported by presented in-culture data. Kenpaullone treatment repaired pathologically attenuated *Kcc2* expression in superficial layers of the spinal cord dorsal horn (SCDH), the principal pain relay in the primary sensory gate of the spinal cord. Importantly, the reversal potential for GABA had become more positive as a result of peripheral nerve injury. Therefore the E-GABA reversal potential was electrically more unstable. This electrophysiological correlate of pain was also repaired toward more negative, and thus electrically more stable, when we treated with kenpaullone; the resulting GABA chloride reversal potential was not different from that pre-injury. Similar analgesic effects were observed with δ-catenin spinal transgenesis to neurons, using AAV9 for delivery of a cassette, containing a short human synapsin promoter to drive a phosphorylation-resistant isoform of δ-catenin, δ-catenin(S276A).

Of several compelling follow-up questions directly emanating from our recent in-depth study (Yeo et al., 2021), we are here addressing two particular issues. We believe that these questions are relevant for translational medical research, and that they are timely because of the significant and increasing unmet medical need in the respective therapeutic arenas. Our questions are rooted in the following rationale.

1) Chemotherapy-induced painful peripheral neuropathy (CIPN) is an important determinant of the quality of life of patients undergoing chemotherapy for malignancy, adding to the enormous disease burden already inflicted by the underlying malignancy (Piccolo and Kolesar, 2014; Fehrenbacher, 2015). In addition, intricately linked to this, CIPN can make chemotherapy protocols less effective because more severe CIPN forces clinicians to cut short ongoing chemotherapy. Thus, in CIPN, can we observe attenuation of *Kcc2* gene expression in the SCDH? In the affirmative case, given that CIPN evokes peripheral small fiber neuropathy with rarefication of peripheral nerve fibers in the skin (Saad et al., 2014; Velasco et al., 2017; Lieber et al., 2018; Bennedsgaard et al., 2020; Timmins et al., 2020), can topical treatment be applied to skin to effectively treat CIPN pain? In the affirmative case, what is the impact of effective topical analgesia for CIPN on *Kcc2* gene expression in the SCDH?
2) As kenpaullone stabilizes the sensory gate in the SCDH by repairing elevated neuronal chloride caused by peripheral nerve injury, does this also apply to the related sensory modality of itch? In other words, does kenpaullone function as an anti-pruritic in chronic pruritus with its peripheral inflammatory injury, suggesting an important role of the SCDH in pruri-transmission, how it is regulated by neuronal chloride, and how its derailed regulation can be repaired by kenpaullone with its *Kcc2* gene expression-enhancing effects?

We are presenting here interesting first-line results in response to these two questions that will form the foundation for dedicated in-depth studies in the future.

## Results and discussion

**Figure 1:**
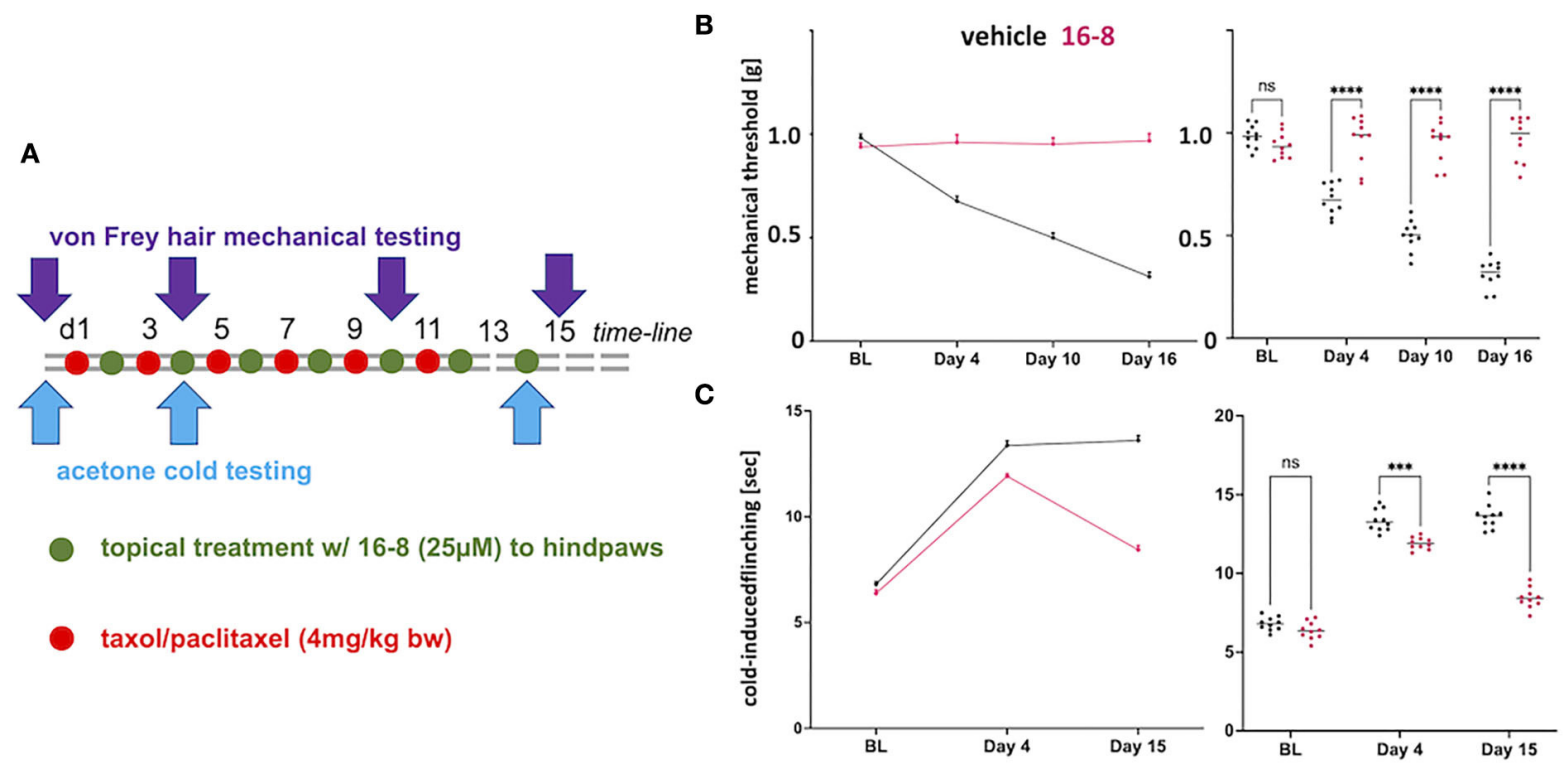
Topical application of compound 16-8 is effective against Taxol-induced nerve pain in mice. **(A)** Shows a schematic illustration of the time-line of the experiment. Red dots stand for i.p injection of Taxol (4 mg/kg), green dots for topical application, to hindpaw skin, of compound 16-8 (25 μM in 50 μL formulation), controls received formulation vehicle only. **(B)** Show the complete protection of development of mechanical allodynia as an effect of topical treatment with compound 16-8. Left-hand line diagram shows time course of average mechanical threshold, as measured using automatic von-Frey-hair testing. Note sensitization in formulation vehicle-treated mice in terms of development of mechanical allodynia. In contrast, mice remained completely normally sensitive when treated with compound 16-8. Purple - compound 16-8-treated, black - formulation vehicle control-treated, upward error bar representing SEM. Right-hand data-cloud diagram show individual data points per mouse, *n* = 10 mice were used per group; note statistically significant difference between compound 16-8-treated (purple) vs. formulation vehicle controls (black), ****p < 0.0001 two-way ANOVA, Sidak's multiple comparison test; lines represent group mean. **(C)** Show the protective effects of topical treatment with compound 16-8 on development of cold allodynia. Diagrams prepared as for **(B)**, with y-axis measurement of cold-avoidance nocifensive behavior. ***p < 0.001, ****p < 0.0001 two-way ANOVA, Sidak's multiple comparison test.

We implemented a mouse preclinical model of CIPN by treating the mice with Taxol (paclitaxel) at 4 mg/kg bw every other day. We then treated these Taxol CIPN-model mice with compound 16-8, a dual TRPV4/TRPA1 inhibitor with sub-micromolar potency against each ion channel target (Kanju and Liedtke, 2016; Kanju et al., 2016). We selected this compound because previous mechanistic investigations of Taxol CIPN revealed significant impact of TRPV4- and TRPA1-mediated calcium influx (Materazzi et al., 2012; Wu et al., 2014), suggested as contributory to calcium overload of peripherally innervating sensory neurons in the dorsal root ganglion (DRG) and trigeminal ganglion (TG) (Sanchez et al., 2020; Sanchez and Ehrlich, 2021). Compound 16-8 was applied to hind-paw skin in a topical formulation for enhanced penetration. Topical treatment was applied every other day. The first topical treatment with compound 16-8 (25 μM in 50 μL) occurred after the first application of taxol. Control mice were treated with a formulation vehicle. The experimental outline is illustrated in Figure 1A. A total of 10 mice per group were examined.

Control-treated mice developed allodynia for mechanical and cold stimulation, when testing for von Frey hair-evoked mechanical withdrawal, using an automatic von Frey apparatus, and when measuring nocifensive behavior in response to cooling evoked by acetone evaporation (Figures 1B,C). In striking contrast to the mechanical and cold allodynia in control mice, we observed a complete absence of mechanical allodynia in compound 16-8 topically treated mice at all three measurement time points, d4, d10, and d15 (Figure 1B). With regards to cold allodynia, the compound 16-8 topically-treated mice first showed signs of attenuated allodynia at d4, thereafter at d15 they were almost back to pre-Taxol normal sensitivity (Figure 1C).

Topical treatment was well-tolerated, without any observable adverse effect. In control mice, treated topically with compound 16-8 or formulation vehicle (*n* = 5 per group), but not sensitized with taxol, there was no detectable compound 16-8 in their systemic circulation at timepoints 2 and 24 h.

**Figure 2:**
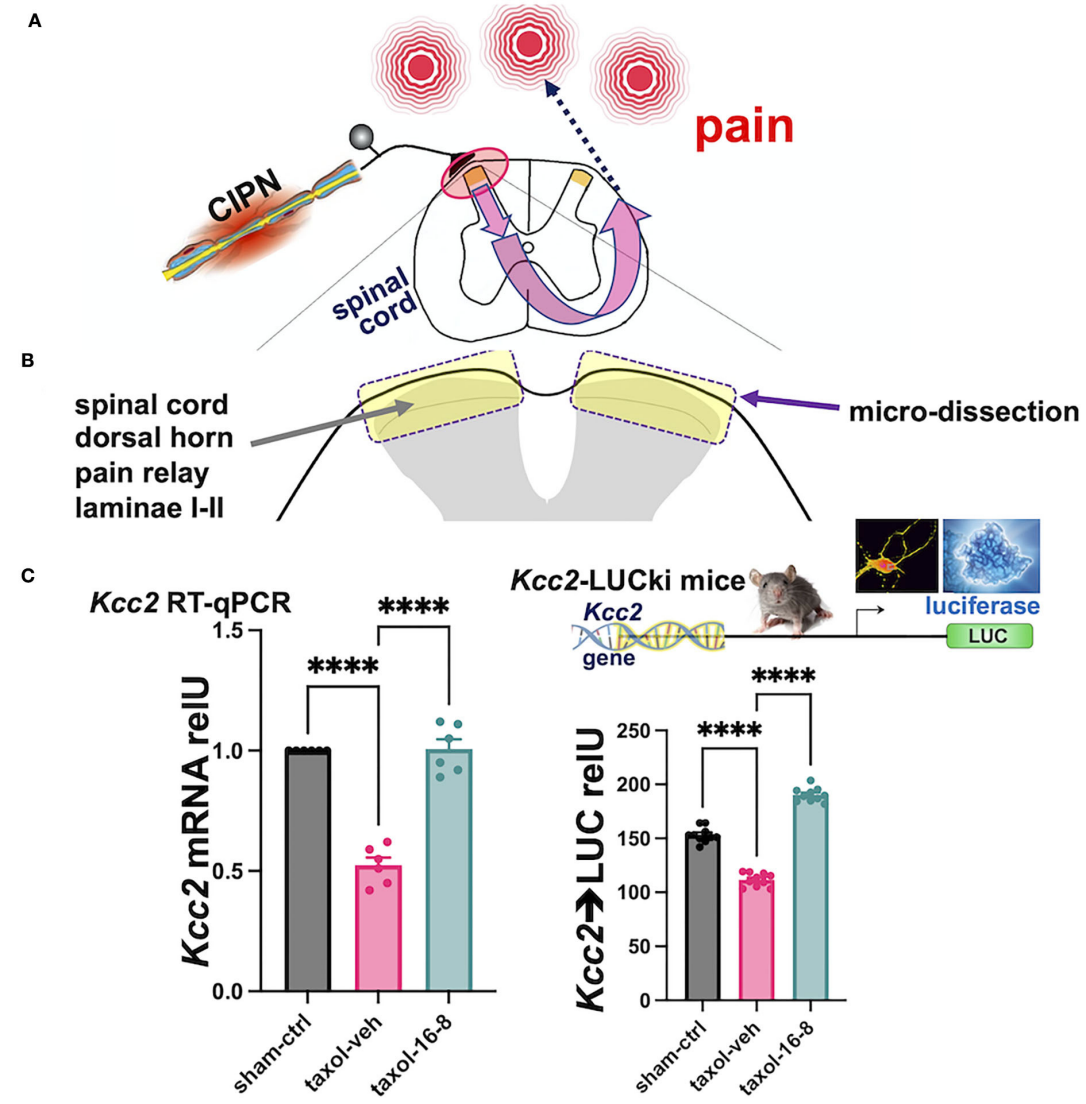
Topical treatment with compound 16-8 repairs damage of the sensory gate function of the spinal cord dorsal horn inflicted by Taxol. **(A)** Shows a schematic representation of the experimental paradigm. Peripheral nerve damage by neurotoxic impact of Taxol sensitizes a central pain circuit, a paradigm widely implemented in preclinical mouse models of human chemotherapy-induced painful peripheral neuropathy (CIPN). **(B)** Shows a schematic representation of our experimental focus on the SCDH and our sampling strategy. SCDH tissue, superficial layers I-II were microdissected as in Yeo et al. (2021), then processed for measurements of *Kcc2* gene expression, as assed by metrics of mRNA abundance of *Kcc2* and *Kcc2* promoter activity. **(C)** These bar diagrams show our results of measuring *Kcc2* gene expression. **(Left)** This bar diagram shows reduced abundance of *Kcc2* mRNA, as measured by RT-qPCR, in response to Taxol. Note significantly reduced levels of *Kcc2* mRNA in formulation vehicle-treated mice (purple) vs. sham (instead of Taxol)-treated mice (black). In compound 16-8-treated mice, notice renormalization of *Kcc2* expression back to sham (instead of Taxol) levels (green). *n* = 6 mice per group, bars represent mean + SEM, ****p < 0.0001, 1-way ANOVA with *post-hoc* Tukey's test. **(Right)** This bar diagram shows results derived from *Kcc2*-LUCki mice, see Methods, for measurements of *Kcc2* promoter activity. Similar to the left-hand panel (same color scheme), activity of the *Kcc2* promoter is significantly reduced when subjecting mice to Taxol, which is renormalized to normal levels in compound 16-8-treated mice. *n* = 10 mice per group, bars represent mean + SEM, ****p < 0.0001, 1-way ANOVA with *post-hoc* Tukey's test.

After behavioral assessment that showed the potent analgesia of peripherally topically applied compound 16-8 (Figure 2A), the animals' spinal cords were sampled and the dorsal horn was microdissected (Figure 2B). From extracted RNA we measured *Kcc2* gene expression *via Kcc2* RT-qPCR. We found a significant reduction of *Kcc2* mRNA abundance in Taxol-treated vs. sham-treated mice (Figure 2C). We then compared compound 16-8-treated mice to formulation vehicle control-treated mice, demonstrating a striking renormalization of *Kcc2* expression to sham-treated (instead of Taxol-treated) levels (Figure 2C). We next asked the question of whether this apparent repair of *Kcc2* gene expression was reflected at the level of the *Kcc2* promoter. For this, we took advantage of a mouse line previously described by our group, namely *Kcc2*-LUCki mice in which the *Kcc2* promoter is driving luciferase (Liedtke et al., 2013; Yeo et al., 2013a, 2021). These mice were conditioned with Taxol (and vehicle for control), then topically treated with compound 16-8 or formulation vehicle, applications as illustrated in Figure 1A. On d15, we performed spinal cord dissection with dedicated dorsal horn microdissection, followed by tissue homogenization and measurement of LUC activity (see Methods). The results were in lockstep with our measurements of *Kcc2* mRNA abundance. We observed a significant reduction of *Kcc2* promoter activity by Taxol conditioning, and then complete repair to non-Taxol levels by peripheral topical treatment with TRPV4/TRPA1 dual-inhibitory compound 16-8.

Thus, we implemented effective peripheral analgesia in a Taxol-CIPN preclinical model. This was accomplished by peripheral-topical administration of TRPV4/TRPA1 dual-inhibitory compound 16-8. Such treatment impacted the pain-transmitting neural circuit in the SCDH by renormalizing *Kcc2* gene expression, which was attenuated by peripheral neural injury caused by exposure to Taxol, an established neurotoxic chemical for peripheral neurons.

We were excited about these results. In terms of the limitations of our findings, we realize that we need to confirm the impact on *Kcc2* expression and KCC2 function *via* electro-physiological interrogation of lamina I-II neurons by measuring their reversal potential for GABA, as we did in our previous study (Yeo et al., 2021). Our findings also are a mandate to identify the mechanisms of action of the TRPV4/TRPA1 dual-inhibitory compound in the periphery, namely its effects on the primary sensory neuron, its support cells, and innervated cells that can tune sensory transduction, such as skin keratinocytes. These items will be the subject of dedicated future studies.

**Figure 3:**
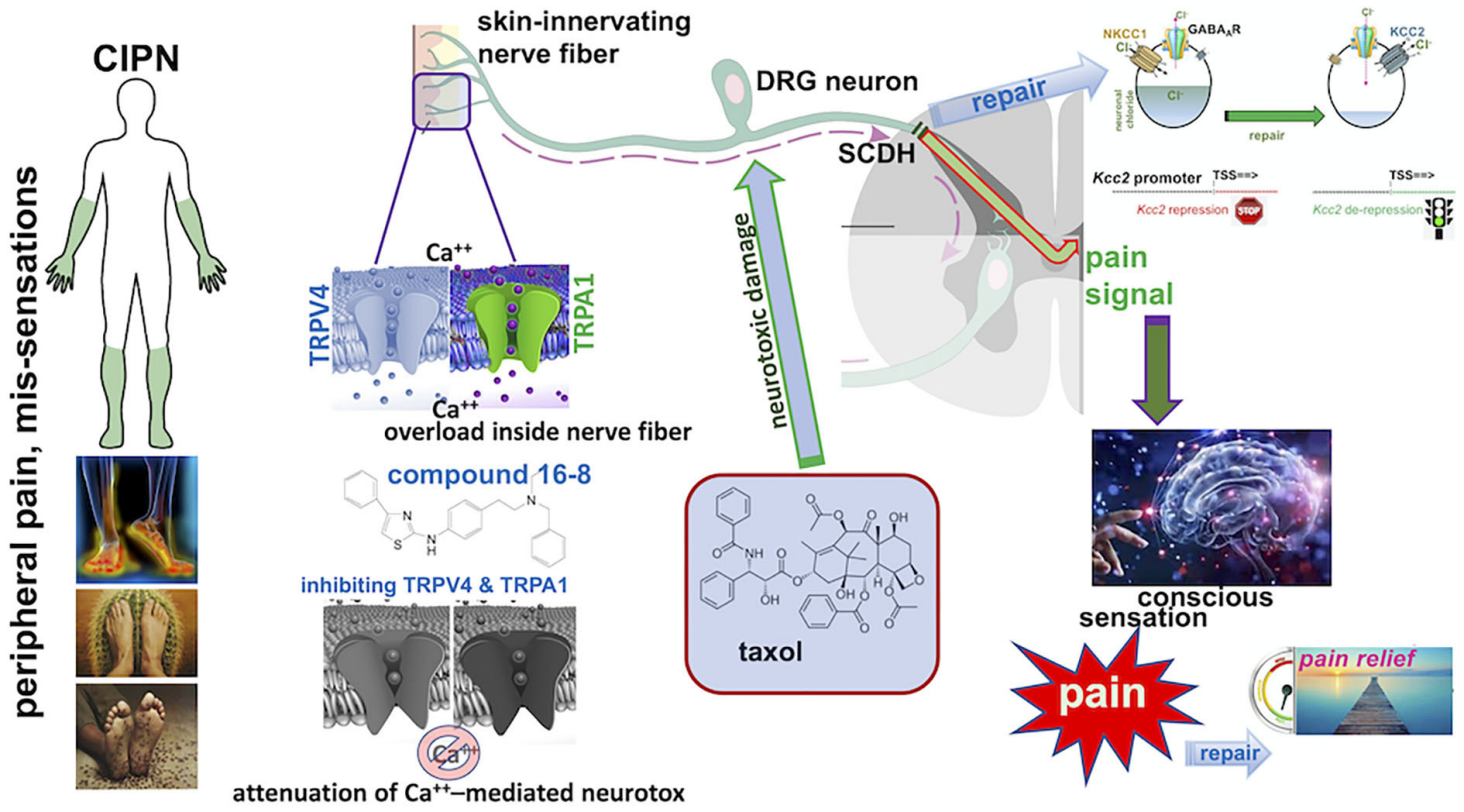
Summary schematics of Taxol CIPN-pain effectively treated with topical compound 16-8 and its beneficial impact on spinal cord dorsal horn (SCDH) neural circuit function. **(Left)** Depiction of the typical “glove and stocking” distribution of chemotherapy-induced painful peripheral neuropathy (CIPN), and frequent subjective symptoms (below). **(Middle)** The pathophysiology of CIPN involves calcium overload in skin-innervating sensory neurons in the dorsal root ganglion (DRG) caused by treatment with neurotoxic chemotherapeutic agents, such as Taxol. **(Right)** Nerve damage to C-type nociceptors in the DRG and their peripheral pain-conducting C-fibers causes sensitization of pain neural circuits which transmit *via* spinal afferents to the brain/cortex, for perception of pain. This involves attenuated expression of *Kcc2* in the spinal cord dorsal horn, thus corrupting the pain neural circuit by rendering its gate function overly excitable and “leaky”. Topical application to skin of compound 16-8 will inhibit TRPV4 and TRPA1 calcium-permeable ion channels in skin-innervating nerve fibers and attenuate the calcium overload-mediated neurotoxicity to DRG neurons. As a result, there is remarkable analgesia in a preclinical mouse model, and also effective repair of attenuated *Kcc2* expression in the SCDH, which resulted from neurotoxic injury of afferents. Upon effective treatment *via* blocking peripheral TRPV4 and TRPA1 channels, *Kcc2* gene expression is renormalized in the SCDH **(Right)**.

Taken together, our findings suggest that (i) CIPN affects the primary pain gate in the SCDH *via* the down-regulated gene expression of *Kcc2*, (ii) peripheral application to the skin of a dual inhibitor of two calcium permeable TRP ion channels, TRPV4 and TRPA1, can repair the behavioral sensitization of CIPN and also *Kcc2* gene expression defects in the SCDH pain gate. These concepts are schematically illustrated in Figure 3.

To learn more about a related sensory submodality, itch, we induced a DNFB contact allergy preclinical model which is known to evoke robust itching, after primary sensitization (systemic), followed by secondary peripheral skin sensitization with the contact allergen, DNFB (Jang et al., 2011; Zhang et al., 2015; Takamori et al., 2018).

**Figure 4:**
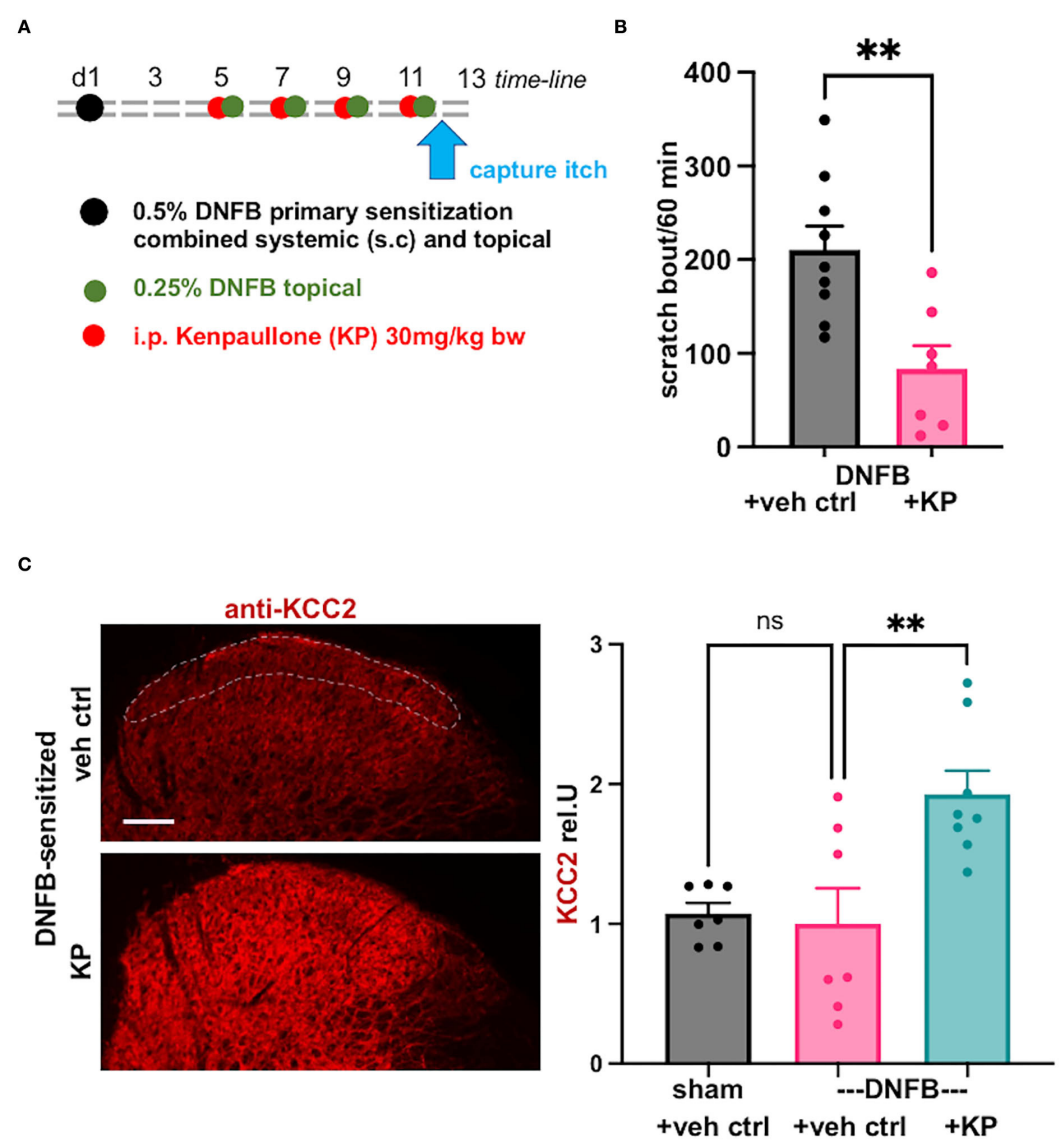
*Kcc2* gene-expression enhancing compound kenpaullone is effective in contact allergy-induced pruritus. **(A)** Shows a schematic overview of the experimental time-line, also see Methods. Videometric recording of scratching behavior as final read-out was conducted on d12. **(B)** This bar diagram shows robust scratching as a result of the DNFB contact sensitization (represented in black), and its significant attenuation by treatment with *Kcc2* gene expression-enhancing compound, kenpaullone (kenpaullone; 30 mg/kg bw; represented in purple), note reduction by >50%. Bars represent group mean + SEM, also individual animal datapoints; *n* = 9 mice for controls, *n* = 7 mice for kenpaullone; **p < 0.01 *t*-test. **(C)** Kenpaullone treatment increases KCC2 expression in the SCDH in DNFB-sensitized mice. Left-hand micrographs: representative KCC2 immuno-staining of the SCDH of cervical segments that innervate the DNFB-treated pruritic area, region-of-interest for densitometric measurement of KCC2 in the SCDH outlined with dotted white line in upper micrograph, focus on Rexed layers I–II because of their relevance for neurotransmission of pruriceptive afferent signals. Scale bar in upper micrograph =100 μm, valid for both panels. Right-hand bar diagrams: kenpaullone [30 mg/kg; treatment protocol see **(A)]** increases KCC2 protein expression in SCDH vs. vehicle control in the DNFB contact allergy pruritus model. Data are represented as mean values ± SEM; *n* = 7-8 mice group; **p = 0.0047, one-way ANOVA.

Indeed, the DNFB-induced contact allergy preclinical model, in our hands, led to robust chronic itching, measured on d12 after initial systemic sensitization (Figure 4A). Treatment with the *Kcc2* gene expression-enhancing kinase inhibitor, kenpaullone, at 30 mg/kg bw, applied after primary sensitization, and directly before each secondary sensitization with DNFB, attenuated scratching behavior with robust effect (>50% reduction) (Figure 4B).

We next queried KCC2 gene expression by specific immunolabeling of the cervical spinal cord, focusing on the segments that innervate the DNFB-sensitized skin, and conducting quantification of the KCC2 signal in laminae-I and -II, as previously conducted (Yeo et al., 2021). We found no significant difference between controls (sham sensitization, vehicle treatment) and DNFB-sensitized animals (DNFB sensitization, vehicle treatment) (Figure 4C). However, there was a tendency for reduced expression of KCC2 in pruritic animals, but also considerable variation in the DNFB-sensitized and vehicle control-treated animals. Importantly, kenpaullone treatment robustly upregulated KCC2 expression, with considerably low variation (Figure 4C). These results suggest that the damage to the inhibitory control of SCDH pruri-transmission is not similar in impact to that affected by pain-causing injury mechanisms, such as nerve constriction, chronic inflammation, and chemo-toxic damage to peripheral nerves. However, in chronic pruritus of DNFB contact sensitization treated with kenpaullone (Figures 4A,B), robust upregulation of KCC2 in the spinal cord dorsal horn was apparent (Figure 4C). This suggests a major contribution of this regulation, which will enhance the inhibitory potency of GABA and glycine as they affect SCDH pruri-transmission, to the strong anti-pruritic effect of kenpaullone.

Given this interesting result, we need to confirm (using a larger experimental sample) and extend these findings with complementary metrics of *Kcc2* gene regulation, such as molecular parameters, also employing physiological measurements such as neuronal chloride levels, *via* chloride imaging, and E-GABA reversal potential, *via* e-phys. This experimental direction is rooted in prior understanding of the central transmission of peripheral itch-causing cues (Koch et al., 2018; Merighi, 2018; Harding et al., 2020).

Also, in view of our recent finding of δ-catenin spinal gene therapy mimicking treatment with kenpaullone (Yeo et al., 2021), whether any anti-pruritic effects of kenpaullone and other GSK3ß-inhibitory compounds can be recapitulated by targeted expression of δ-catenin in the SCDH. If this could be accomplished it would be of great interest which molecularly distinct neuronal lineage in the SCDH were critical for this effect.

Both approaches taken, the CIPN pain model and DNFB itch model, also raise the question of the possible contribution of enhanced presynaptic inhibition in the SCDH (Betelli et al., 2015; Gradwell et al., 2020) to the observed effects of analgesia, accomplished by a topical treatment to skin that ultimately targets skin-innervating DRG sensory neurons, and to the observed antipruritic effect of kenpaullone. Obviously, a chloride-lowering effect of enhanced expression of *Kcc2* in the distal-central axon of the primary afferent would explain both, yet this was not interrogated here.

Given the focused, thus limited, scope of the current study, vs. the extensive scope of these highly relevant questions, the latter will be the subject of future investigations.

Taken together, our current results suggest that pruri-transmission in the SCDH under the condition of peripheral chronic allergic-inflammatory injury can be impacted by augmenting the inhibitory tone of GABA and glycinergic transmission. This in turn was accomplished by upregulating the gene expression of *Kcc2* with kenpaullone, presumably also feasible with other *Kcc2* gene expression-enhancing approaches such as other GSK3ß-inhibitors, or directed δ-catenin over-expression. This would represent an attractive new approach to the attenuation of chronic pruritus.

## Materials and methods

### Animals

As described in Yeo et al. (2021), C57BL/6J male mice (10–12 weeks old) were obtained from The Jackson Lab (Bar Harbor, ME). *Kcc2-*LUCki mice were generated by the former Liedtke-Lab at Duke University and continued internally. All animal procedures were approved by the Duke University IACUC and carried out in accordance with the NIH's Guide for the Care and Use of Laboratory Animals. Mice were housed in a temperature- and moisture-controlled environment, 12/12 h light-dark cycle with laboratory mouse certified food available *ad libitum* as well as water.

### Taxol preclinical CIPN model

Taxol (paclitaxel; SIGMA), in a vehicle of ethanol and Cremophor EL (1:1), was diluted in saline before use. For injection into mice, each mouse received intraperitoneal injections of Taxol every other day, with a total of six injections (Figure 1A), using an injection volume of 100 uL.

### Topical treatment with compound 16-8; formulation vehicle

Compound 16-8 was synthesized as in Kanju et al. (2016). Its purity was determined by HPLC and liquid chromatography/mass spectrometry (LC-MS/MS) (Kanju et al., 2016). In total of 10 mM stock solutions were prepared in DMSO. The stock solution was diluted to 25 μM in a formulation vehicle, composed as follows.

Carbopol-980, 50 mg/10 mL, Carbopol Ultrez-10, 150 mg/10 mL (both carbopol from Lubrizol, Wickliffe OH), ethanol 1 mL/10 mL, isopropanol 1 mL/10 mL, water up to 10 mL.

We used this vehicle based on earlier descriptions (Buyuktimkin et al., 2014; Liedtke et al., 2015) to enhance epidermal penetration so that subepidermial nerve fibers could be reached.

### Determining concentration of compound 16-8 in serum

Concentration of compound 16**-**8 was determined in serum by mass-spectrometric analytical chemistry methods as described in Kanju et al. (2016). Positive controls were carried along and the assay had a threshold of detection of <0.1 nM, which is >4000x the dilution of the ED50 of compound 16-8 against either of its targets, TRPV4 and TRPA1.

### Behavioral metrics

#### von Frey test

Mechanical pain behavior was assessed using electronic von Frey filaments (Ugo Basile, Italy). Mice were habituated to the testing environment for at least 2 days before baseline testing. Mice were placed in individual cages with a mesh floor. The von Frey filaments of differing forces were applied perpendicularly to the middle of the plantar surface of the hind paw and the withdrawal responses after the stimulation were measured 3 times and averaged.

#### Acetone cold test

Mice were placed in individual cages equipped with video-cam monitoring and were allowed to acclimatize for 30 min before testing. Cotton buds dipped in cold acetone was applied to both hind paws and the time of paw flinching in response to evaporative cooling was recorded for the next minute. The flinching responses were measured four times and averaged, with a 5-min wait interval between trials.

### SCDH microdissection

Lumbar spinal cords were microdissected directly post-euthanasia and submerged in ice-cold artificial cerebrospinal fluid (aCSF). Under 50x magnification, using a Zeiss dissecting microscope, L5 and L6 segments were cross-sectioned with razor blades. Next, spinal cord dorsal horn superficial layers were removed, bilaterally, using iridectomy scissors. Microdissected tissue was then immersed in RNAzol lysis buffer for *Kcc2* RT-qPCR or in aCSF for subsequent luciferase assays.

#### Kcc2 RT-qPCR

As described in Liedtke et al. (2013) and Yeo et al., 2013a, 2021, total RNA was extracted from the microdissected lumbar SCDH. A measure of 1 μg of total RNA was reverse transcribed using oligodT and subjected to RT-qPCR using primers specific for *Kcc2* and using neuronal β_III_-tubulin as the normalization gene. The ΔΔCt method was used to compute relative *Kcc2* gene expression.

*Kcc2* primers:

Forward: 5′CTGACGGACTGCGAG GACGG3′ Reverse: 5′GGCTGGTGTCCATCTCCTCCTCAA3′ neuronal β_III_-tubulin primers:

Forward: 5′CCTGCCTTTTCGTC TCTAGCCGC3′ Reverse: 5′GCTGATGACCTCCCAGAACTTGGC3′.

### *Kcc2* LUC measurements from *Kcc2*-LUCki mice

*Kcc2*-LUCki mice were used, as described in Liedtke et al. (2013) and Yeo et al. (2013a, 2021). They were subjected to the same treatment protocol as shown in Figure 1A. Spinal cords - SCDH were microdissected post-euthanasia, as described above. Bilateral L5-L6 SCDH were pooled for each animal and homogenized in 150 μl lysis buffer (Targeting Systems CA, USA, cat.# CLR1), also using syringes and needles 15G−22G. LUC activity was measured with a Red-Luciferase Assay kit (Targeting Systems cat.# FLAR) following the manufacturer's instructions. A Veritas microplate luminometer was used to measure luminescence; 60 μL SCDH lysate was used and 40 μL substrate was injected per well.

### Spinal cord immuno-histochemistry

Immuno-histochemistry of the cervical spinal cord was conducted as in Yeo et al. (2021), using KCC2-specific antibody, Millipore (07-432), at 1:2000 dilution.

### DNFB chronic contact allergy model: Metrics of itch

An allergic contact dermatitis model was established by applying 1-fluoro-2, 4-dinitrobenzene (DNFB) onto the skin on the back of the neck, as described previously (Zhang et al., 2022). In brief, mice were sensitized with s.c. injection of 50 μL of 0.5% DNFB solution [in a mixture of acetone:olive oil (4:1)], followed by the topical application of 100 μL to a 1 cm^2^ area of shaved abdomen skin on day 1. Mice were then challenged with 100 μL of 0.25% DNFB solution by painting the nape of neck on day 5, 7, 9, and 11. To examine the effect of kenpaullone on scratching behavior-induced by DNFB, mice received an intraperitoneal (i.p.) injection of either kenpaullone (30 mg/kg body weight) or the vehicle (5% DMSO and 5% Tween-80 in normal saline) 20 min before each DNFB challenge on day 5, 7, 9, and 11. On day 12, mice were allowed to acclimate to a plexiglass chamber equipped with video camera for at least 30 mins before the behavior test. Scratching behavior was recorded by a Panasonic video camera for a 1-h observation period. Hindlimb scratching behavior directed toward the treatment area at the nape of neck was measured as scratching behavior. One scratch bout was defined as a lifting of the hindlimb toward the treatment area and then a replacing of the limb back to the floor or to the mouth for licking, regardless of how many scratching strokes take place between those two movements. Behavioral analysis was conducted by an observer blinded to treatment procedure.

### Statistics

As described in Yeo et al. (2021), all data are expressed as mean + SEM. Statistical analysis was conducted using Graphpad Prism9.1 software. Differences between groups were evaluated using two-tailed, unpaired Student's *t* test (experimental against sham/vehicle control), or in the case of multiple groups, one-way ANOVA followed by *post-hoc* Tukey or Dunnett test, following the guidance of the GraphPad program. When applying two-way ANOVA for multiple group comparison, we used the *post-hoc* Sidak test, following the guidance of the GraphPad program. The criterion for statistical significance was *p* < 0.05.

## Data availability statement

The original contributions presented in the study are included in the article/supplementary material, further inquiries can be directed to the corresponding author/s.

## Ethics statement

The animal study was reviewed and approved by Duke University School of Medicine IACUC.

## Author contributions

All authors listed have made a substantial, direct, and intellectual contribution to the work and approved it for publication.

## Funding

This study was supported by NIH grants to WL (NS066307), YC (DE027454), and by the Michael Ross Haffner Foundation (Charlotte NC) to WL. Duke Neurology provided internal support to WL.

## Conflict of interest

Author WL is a full-time executive employee of Regeneron Pharmaceuticals, Tarrytown NY. Author WL co-founded TRPblue Inc. (Durham NC) in 2017. The remaining authors declare that the research was conducted in the absence of any commercial or financial relationships that could be construed as a potential conflict of interest.

## Publisher's note

All claims expressed in this article are solely those of the authors and do not necessarily represent those of their affiliated organizations, or those of the publisher, the editors and the reviewers. Any product that may be evaluated in this article, or claim that may be made by its manufacturer, is not guaranteed or endorsed by the publisher.
